# Effect of Cocoa and Its Flavonoids on Biomarkers of Inflammation: Studies of Cell Culture, Animals and Humans

**DOI:** 10.3390/nu8040212

**Published:** 2016-04-09

**Authors:** Luis Goya, María Ángeles Martín, Beatriz Sarriá, Sonia Ramos, Raquel Mateos, Laura Bravo

**Affiliations:** 1 Departamento de Metabolismo y Nutrición; Instituto de Ciencia y Tecnología de Alimentos y Nutrición (ICTAN – CSIC), José Antonio Novais, 10, Madrid 28040, Spain; amartina@ictan.csic.es (M.Á.M.); beasarria@ictan.csic.es (B.S.); s.ramos@ictan.csic.es (S.R.); raquel.mateos@ictan.csic.es (R.M.); lbravo@ictan.csic.es (L.B.); 2 Centro de Investigación Biomédica en red de Diabetes y Enfermedades Metabólicas Asociadas (ISCIII), Madrid 28029, Spain

**Keywords:** flavanols, chocolate, cardiovascular disease, colon cancer, colon inflammation, anti-inflammatory flavonoids, anti-inflammatory-polyphenols

## Abstract

Chronic inflammation has been identified as a necessary step to mediate atherosclerosis and cardiovascular disease and as a relevant stage in the onset and progression of several types of cancer. Considerable attention has recently been focused on the identification of dietary bioactive compounds with anti-inflammatory activities as an alternative natural source for prevention of inflammation-associated diseases. The remarkable capacity of cocoa flavanols as antioxidants, as well as to modulate signaling pathways involved in cellular processes, such as inflammation, metabolism and proliferation, has encouraged research on this type of polyphenols as useful bioactive compounds for nutritional prevention of cardiovascular disease and cancer. Data from numerous studies suggest that cocoa and cocoa-derived flavanols can effectively modify the inflammatory process, and thus potentially provide a benefit to individuals with elevated risk factors for atherosclerosis/cardiovascular pathology and cancer. The present overview will focus on the most recent findings about the effects of cocoa, its main constituents and cocoa derivatives on selected biomarkers of the inflammatory process in cell culture, animal models and human cohorts.

## 1. Introduction

Inflammation is a protective physiological response of body tissues to harmful stimuli, such as pathogens, damaged cells, or irritants, that involves immune cells, blood vessels, and molecular mediators [1]. The purpose of inflammation is to eliminate the initial cause of cell injury, clear out necrotic cells and tissues damaged from the original insult and the inflammatory process, and to initiate tissue repair. Although inflammation is normally closely regulated by the body, chronic inflammation may lead to a host of diseases, including the two most lethal pathologies of our time, cardiovascular disease and cancer [1].

### 1.1. Inflammation and Cardiovascular Dysfunction

Chronic inflammation has been identified as a necessary step to mediate atherosclerosis and cardiovascular disease [2,3]. Multiple risk factors for atherosclerosis and cardiovascular disease act in a coordinated or synergistic way through one or more inflammatory pathways. Risk factors appear to act on three cell types that coordinate their action to influence cardiovascular dynamics, function, and structure. These cell types include: (i) endothelial cells that line the vascular lumen and control the transcellular flow of nutrients, hormones and immune cells, and regulate vascular tone and blood flow; (ii) smooth muscle cells that maintain vascular tone and structure; and (iii) immune cells, including monocytes/macrophages and T lymphocytes, which defend the endothelium and smooth muscle cells from chemical and biological insults [3]. The disruption or over-expression of the coordinated activities of these cells can lead to chronic inflammation and cardiovascular disease. Risk factors for cardiovascular disease with a pro-inflammatory component include LDL cholesterol, smoking, elevated blood sugar, hypertension, diabetes, infections, oxidant damage, interleukin-6 (IL-6) and C-reactive protein (CRP). In addition, these inflammatory risk markers can react synergistically to increase relative risk [3]. Thus, inflammatory mediators play a key role in the pathology of atherosclerosis, starting from the initial phases of leukocyte recruitment, and finishing with the eventual rupture of the atherosclerotic plaque [4]. Indeed, inflammation itself is recognized as a cardiovascular risk factor [2,5], and atherosclerosis and cardiovascular pathologies are readily recognized and treated as inflammatory diseases [6]. Consequently, suppression of inflammation by chemo-preventive compounds seems to be an attractive strategy to regulate the development and progression of cardiovascular disease.

### 1.2. Inflammation and Cancer

Chronic inflammation has also been recognized as a relevant step in the onset and progression of several types of cancer. During cancer progression, chronic inflammation is causally linked to carcinogenesis and acts as a driving force in the premalignant and malignant transformation of cells [7]. The milestone for the process of chronic inflammation is an increase in the activity of the pro-inflammatory enzymes cyclo-oxygenase-2 (COX-2) and inducible nitric oxide (NO) synthase (iNOS) which creates a microenvironment contributing to the development of preneoplastic lesions [8]. More significantly, inhibition of these two enzymes has shown protective effects against tumor development in different animal models, suggesting that they are crucial targets for tumorigenesis [8]. It has been suggested that an inflammatory microenvironment can increase mutation rates, in addition to enhancing the proliferation of mutated cells [7]. Activated inflammatory cells are sources of reactive oxygen species (ROS) that are capable of inducing DNA damage and genomic instability. Alternatively, inflammatory cells may use cytokines such as tumour necrosis factor alpha (TNF-α) to stimulate ROS accumulation in neighboring epithelial cells [7,8]. Furthermore, Grivennikov and colleagues [9] demonstrated that the redox-sensitive eukaryotic transcription factor nuclear factor kappa B (NF-κB), that regulates the expression of iNOS and COX-2, is constitutively activated in neoplastic cells and may constitute a dangerous association in the development of cancer. The inflammatory microenvironment is completed by pro-inflammatory cytokines produced by immune cells. Among these cytokines, the pro-tumorigenic function of TNF-α and IL-6 is well established. The role of TNF-α and IL-6 as master regulators of tumor-associated inflammation and tumorigenesis makes them attractive targets for adjuvant treatment in cancer [10]. Therefore, the use of chemo-preventive compounds that suppress inflammation seems to be a useful strategy to control the development and progression of several cancers.

### 1.3. Cocoa Flavonoids as Anti-Inflammatory Compounds

Considerable attention has recently been focused on the identification of dietary compounds with anti-inflammatory bioactivity as an alternative natural source for prevention of inflammation-associated diseases [11]. During the last decade, a growing number of studies have demonstrated that flavanols, a sub-family of the flavonoid family of polyphenols, show the capacity to modulate inflammation, as well as other major metabolic and immunological pathways [12]. The molecular mechanisms underlying their chemo-preventive effects have been associated with their antioxidant capacity, as well as the modulation of signalling cascades and expression of genes involved in the regulation of cell proliferation and apoptosis and the suppression of chronic inflammation [13]. Indeed, the capacity of flavanols to modulate signalling pathways involved in cellular processes such as inflammation, metabolism, proliferation and apoptosis has encouraged research on this type of polyphenols as useful bioactive compounds for nutritional prevention of cardiovascular disease and cancer.

Flavanols or catechins are present in many fruits and vegetables, but mainly in tea, wine and cocoa [14,15,16]. Cocoa is a rich source of antioxidants; in a study that measured the total concentration of redox compounds in 1113 different foods, out of the 50 foods with the highest antioxidant capacity, five were cocoa based [17]. Indeed, cocoa has the highest flavanol content of all foodstuffs on a weight basis and is a significant contributor to the total dietary intake of flavonoids [14,15,16]. Cocoa contains high amounts of the flavonoids (−)-epicatechin (EC), (+)-catechin and their dimers procyanidins B2 (PB2) and B1, although other polyphenols such as quercetin, isoquercitrin (quercetin 3-*O*-glucoside), quercetin 3-*O*-arabinose, hyperoside (quercetin 3-*O*-galactoside), naringenin, luteolin and apigenin have also been found in minor quantities [18]. Cocoa powder is also a rich source of fiber (26%–40%), proteins (15%–20%), carbohydrates (about 15%) and lipids (10%–24%) and it contains minerals and vitamins [19]. Cocoa and derivatives, especially chocolate, are widely consumed worldwide, due to the highly attractive organoleptic characteristics. Indeed, cocoa products constitute a larger proportion of the diet of many individuals, than green tea, wine, or soy beans [20,21]. The mean intake of catechins and procyanidins estimated for USA is higher than the estimated intake of other flavonoids [22]. Chocolate consumption contributed 2–5 mg of daily catechin intake out of an estimated total of 50 mg per day in a report from the Netherlands [20]. Finally, it has been estimated that cocoa products account for 10% of the total antioxidant capacity of Spanish dietary intake [21].

### 1.4. Bioavailability and Distribution of Cocoa Flavanols

Health effects derived from cocoa flavonoids depend on their bioavailability (absorption, distribution, metabolism, and elimination), which is also influenced by their chemical structure [23]. In this regard, different studies have shown the absorption of catechin, EC and dimeric procyanidins, after the intake of different cocoa-derived products by animals and humans [24,25]. In particular, monomeric flavonoids are absorbed in the small intestine and together with their metabolites (*i.e.*, methylated, sulfated, and glucuronidated compounds), which may retain bioactivity, are rapidly detected in the plasma at concentrations in the range of nM to μM [26,27,28] and urine [29]. Absorbed flavanols are extensively distributed and can be found in lymphoid organs such as the thymus, spleen and mesenteric lymphoid nodes, as well as in the liver and testes at different concentrations [30]. In contrast, procyanidins (dimers and trimers) and large proanthocyanidins appear to be 10–100-fold less absorbed [23,31]. Therefore, their beneficial effects would be mainly circumscribed to the gastrointestinal tract where they may have an important local function [19,32]. Furthermore, oligomers and polymers of flavanols that are not absorbed through the gut barrier are metabolized by the intestinal microbiota into various phenolic acids of low molecular weight, which are more bioavailable, and might be well absorbed through the colonic mucosa [25,30]. Interestingly, recent findings have revealed that some of these microbial metabolites derived from cocoa consumption also hold biological properties [33,34].

### 1.5. Cocoa Flavanols in the Prevention of Cardiovascular Disease and Cancer

As stated above, because of their anti-inflammatory capacity, flavanols could be useful in the prevention and treatment of high-prevalence disorders, such as cardiovascular disease and cancer. Data from numerous studies suggest that cocoa-derived flavanols can effectively modify the inflammatory process [2,12,19], and thus, potentially provide a benefit to individuals with elevated risk factors for atherosclerosis/cardiovascular pathology and cancer. The implication of the anti-inflammatory effect of flavanols on endothelial and cardiovascular function has been extensively reviewed by Cooper *et al.* [12] and Selmi *et al.* [35] and, more recently, in this same journal, by Khan *et al.* [2]. On the other hand, studies performed in animals have demonstrated that cocoa and its main phenolic components, probably through their antioxidant and/or anti-inflammatory capacity, may prevent and/or slow down the initiation-progression of different types of cancers such as cancer of the prostate, liver, colon, leukemia, *etc.* [36]. In addition, several human intervention studies have reported some favorable changes in biomarkers assessing antioxidant and anti-inflammatory status that could have a role in the onset and incidence of human tumorigenesis, all of which have been recently reviewed [36,37]. The present article will review some recent findings on the anti-inflammatory effects of cocoa and cocoa flavanols on cell and tissue function related to cardiovascular disease and cancer plus additional results regarding the anti-inflammatory capacity of cocoa flavanols on other cell types and tissues not previously reviewed. Data will be separated into three different sections regarding the experimental models used for the study, cell culture, animal experimentation and human studies.

## 2. Studies of Cell Culture

Cell culture studies constitute a useful tool to elucidate the molecular mechanisms of action of flavanols including those related to inflammatory processes. It should be mentioned that both free flavanols and their metabolites circulate in blood, and some flavanol metabolites have shown to have a remarkable biological activity [33,34], indicating that their synergic effect to that of pure compounds should not be ruled out. Moreover, intracellular and bound phenolic metabolite concentrations can be higher than plasma levels, and can be significantly bioactive even when plasma concentrations are in the nM range [38]. These crucial points should be considered when discussing flavanol doses used in cell culture studies and the potential value of their extrapolation to a whole-organism situation.

The physiological process of inflammation involves key inflammatory mediators, such as NF-κB, TNF-α, COX-2 and lipoxygenases (LOX). These proteins are closely related to endothelial dysfunction [2], as well as to cell proliferation, anti-apoptotic activity, angiogenesis and metastasis [39]. A specific effect of cocoa flavanols on the *in vitro* (cell-free) activity of some of these mediators has been reported. Thus, cocoa flavonoids were shown to inhibit enzyme activity of several mammalian LOX, involved in arachidonic acid metabolism and the synthesis of several inflammatory mediators [40]. The biological role of 5-LOX is closely connected with the biosynthesis of leukotrienes, and the inhibition of human 5-LOX by cocoa flavonoids suggests anti-leukotriene actions of these compounds, which may confer some anti-inflammatory, vasoprotective, and anti-bronchoconstrictory capacity [40]. 

The production of pro-inflammatory cytokines by immune cells is a critical step in the establishment and maintenance of an inflammatory status, and is, therefore, a primary target for putative anti-inflammatory interventions [35]. Thus, a number of cell culture studies have focused on immune defense system blood cells, such as platelets, macrophages, lymphocytes, peripheral mononuclear, *etc.*, and the results of these studies have been systematically reviewed in a recent article by Khan and colleagues [2]. Reports on cells derived from other tissues are not as frequent and this topic requires further coverage. In particular, cell culture studies have demonstrated that cocoa phenolic compounds exhibited a variety of potential anti-inflammatory effects in intestinal cells [41,42,43,44], which may contribute to their cancer chemo-preventive activity (Table 1). Thus, cocoa procyanidins have been shown to inhibit TNFα-induced NF-κB activation, iNOS, and cell oxidant increase in Caco-2 cells [41]. These effects occurred because cocoa hexameric procyanidins can inhibit binding of TNF-α to its receptor and the subsequent NF-κB activation [41]. More recently, high molecular weight polymeric procyanidins from cocoa decreased IL-8 in human colon cancer HT-29 cells stimulated with TNF-α [42]. A cocoa phenolic extract inhibited the inflammatory mediator prostaglandin E2 in human intestinal Caco-2 cells [43]. In the same colonic cell line, a comparable cocoa phenolic extract significantly reduced the increase in inflammatory markers such as IL-8 secretion, COX-2 and iNOS expression induced by the pro-inflammatory agent TNF-α [44]. In this study, the cocoa phenolic extract selectively decreased the nuclear translocation of NF-κB induced by TNF-α, indicating that this pathway could be an important mechanism contributing to the reduction of intestinal inflammation. In mouse epidermal cells, cocoa polyphenols inhibited TNF-α-induced phosphorylation of protein kinase B (PKB/AKT) and extracellular-regulated kinases (ERK) and suppressed the TNF-α-induced mitogen activated protein kinase-1 (MEK1) activity and the phosphatidylinositol-3-kinase (PI3K) activity, suggesting a chemopreventive potential against pro-inflammatory cytokine-mediated skin cancer and inflammation [45]. Similarly, inhibition of nitrite and TNF-α production has been reported for (+)-catechin in lipopolysaccharide (LPS)-stimulated macrophages [46] and for EC in murine aorta endothelial cells [47]. Furthermore, NF-κB activation was reduced in RAW 264.7 murine macrophage cells treated with a polyphenol-enriched cocoa extract [48] and treatment of peritoneal macrophages cultured *ex vivo* with the same cocoa extract reduced IL-6, IL-1β, and TNF-α induced by LPS [48]. All these results have been summarized in Table 1.

Additionally, anti-inflammatory effects of cocoa polyphenols may also indirectly result from their remarkable antioxidant capacity. Antioxidant activity is mediated by the ability to scavenge free oxygen and nitrogen species, abrogating the pro-inflammatory activity of ROS-generating enzymes such as COX, LOX, and iNOS [36,37,39,44]. Cocoa flavanols also stimulate activities of crucial antioxidant enzymes such as catalase, superoxide dismutase, glutathione peroxidase (GPx), glutathione reductase (GR) and glutathione-S-transferase (GST) [51]. Indeed, antioxidant capacity of EC to protect monocytes from oxidative stress is crucial for an efficient anti-inflammatory effect of cortisol [52]. Essential biological activities related to the antioxidant effects of cocoa flavonoids have been recently reviewed by Martin *et al.* [36].

Not all cell culture studies have, however, supported the anti-inflammatory effect of cocoa and its polyphenols (Table 1). In a recent study performed in differentiated Caco-2 cells, cocoa polyphenols neither affected IL-6 and IL-8 production, nor enhanced high density lipoproteins (HDL) functionality after LPS-induced inflammation [49]. Furthermore, in liver-derived HepG2 cells, EC transiently stimulated the NF-κB pathway by increasing NF-κB (p65) levels and IκB kinase, and enhancing NF-κB nuclear translocation and DNA binding activity [50]. But in this case, induction of the redox-sensitive transcription factor NF-κB was associated to that of ERK, which is involved in the control of hepatic cell survival and proliferation, pointing out to the role of EC in the promotion of cell protection and survival pathways [50]. The study of the intrinsic mechanisms involved in inflammation as well as the evaluation of biomarkers of the cellular antioxidant defence system in such conditions could greatly help to identify potential strategies for the prevention or disappearance of inflammation-related diseases. In this line, nutritional sciences can benefit from cell culture models where the molecular mechanisms involved in the biological effect of potential antiinflammatory compounds can be accurately assayed. A critical point for the potential extrapolation of data from cell culture to a whole-organism situation in humans is the use of realistic doses of the chemo-protective compounds. After normal dietary intakes, polyphenols and their metabolites appear in the circulatory system at nM–μM concentrations, so they are the most appropriate doses for *in vitro* studies [26,27].

## 3. Studies of Experimental Animals

Animal studies offer an excellent opportunity to assess the contribution of the physiological effects of consumption of cocoa and cocoa components in different models of inflammation. Interestingly, addition of cocoa to experimental diets has regularly shown high acceptation by the animals and no toxicity even in chronic studies for more than 100 weeks [53]. More recently, no evidence of inflammatory processes or carcinogenicity was found in chronic studies with dietary doses of 5% [54] and 12% [32,44] of cocoa powder in rats. As a matter of fact, cocoa administration has been shown to reduce inflammation induced by adjuvant arthritis in rats [55]. Thus, administration for seven days of a high-cocoa diet (4.8 g/kg/day) reduced the production of the pro-inflammatory cytokines IL-6 and TNF-α, as well as of NO and ROS, in rat peritoneal macrophages *ex vivo* [55].

### 3.1. Cocoa and Cardiovascular Disease in Animal Models

A number of animal studies have supported the anti-inflammatory effect of cocoa-enriched diets with a potential impact in cardiovascular health (Table 2). Hence, cocoa supplementation (8%) for 10 weeks significantly decreased the plasma levels of the pro-inflammatory mediator IL-6 and the expression of several pro-inflammatory genes in mice submitted to a high fat diet [56]. Also, in high-fat fed mice, the same group has reported that dietary cocoa reduces adipose tissue inflammation [57]. In this study, cocoa supplementation decreased adipose tissue mRNA levels of TNF-α, IL-6, iNOS, and nuclear protein levels of NF-κB. Finally, cocoa treatment reduced the concentration of arachidonic acid and protein levels of the eicosanoid-generating enzymes, adipose-specific phospholipase A2 and COX-2 in the adipose tissue [57]. Also in mice submitted to a high fat diet, cocoa treatment counteracted lipid storage in the liver and improved the lipid-metabolizing activity and oxidative stress defences, a process that seems to be mediated by the activation of peroxisome proliferator-activated receptor α (PPARα) [58]. The flavanol fraction of cocoa powder suppressed changes associated with early-stage metabolic syndrome in high-fat diet-fed rats [59]. In addition to their hypotensive effects, cocoa flavanols enhanced thermogenesis and lipolysis, and consequently reduced white adipose tissue weight gain in response to high-fat diet feeding [59]. In another study, a cocoa polyphenol extract suppressed cardiovascular inflammation and oxidative stress in a mice model of experimental myocarditis [60]. Treatment of these mice with the cocoa polyphenol extract, reduced mRNA expressions of IL-1β, IL-6, E-selectin, vascular cell adhesion molecule (VCAM)-1, as well as the phosphorylation of NF-κB p65 in heart [60]. Finally, administration of the main cocoa flavanols, catechin and EC, reduced arterial blood pressure in spontaneously hypertensive rats, an effect which was not observed in normotensive animals [61]. However, not all animal studies have unequivocally confirmed the anti-inflammatory effect of cocoa flavanols, since a high-polyphenol chocolate administration showed no effects on plasma levels of VCAM-1 and E-selectin in ApoE3-Leiden mice fed with a high cholesterol diet [62]. These results have been summarized in Table 2.

### 3.2. Cocoa and Colon Inflammation and Cancer in Animal Models

Cocoa and its flavanols have shown a potential preventive effect against several types of cancer, including mammary, lung, prostate, liver and colon [36]. However, due to the multiple biological activities reported for flavanols, *i.e.*, antioxidant, anti-proliferative, pro-apoptotic, anti-inflammatory, *etc.*, the specific mechanism of action responsible for the anticancer effect might not be unique and could differ depending on the cancer type. Most of the studies of cocoa flavanols on animal models of cancer have not explored parameters of inflammation, precluding establishing a direct correlation between the anti-inflammatory capacity and cancer prevention. The only cancer type where the anti-inflammatory ability of cocoa flavanols has revealed a crucial role in the prevention of neoplastic lesions is colorectal cancer [36,37].

#### 3.2.1. Cocoa and Colon Inflammation

Cocoa has a high concentration of procyanidins that are poorly absorbed in the intestine where they may have an important antioxidant and anti-inflammatory local function; therefore, they could have a major role in the prevention of the onset and development of inflammatory bowel disease and colorectal cancer. Certainly, most of the published works support the capability of cocoa polyphenols to effectively suppress the production of cytokines and adhesion molecules that promote inflammation in the colon (Table 3) [63]. For instance, in a mouse model for ulcerative colitis, one of the main manifestations of the inflammatory bowel disease, oral administration of a polyphenol-enriched cocoa extract significantly reduced the severity of the colon inflammation, as well as decreased crypt damage and leukocyte infiltration in the mucosa [48]. Similarly, in a rat model for ulcerative colitis by administration of dextran sulfate sodium (DSS), a diet enriched with a 5% of cocoa powder for two weeks showed anti-inflammatory potential because it down-regulated serum TNF-α, colon iNOS activity and decreased colon cell infiltration [54]. More recently, it has been observed that dietary cocoa inhibits colitis-associated cancer in a mouse model of azoxymethane (AOM)/DSS-induced chronic inflammation by activating the nuclear factor (erythroid-derived 2)-like 2 (Nrf2) pathway [64] and suppressing the IL-6/Signal transducer and activator of transcription (STAT)-3 pathway [65]. 

#### 3.2.2. Cocoa and Colon Cancer

As stated above, the anti-inflammatory local function of cocoa procyanidins could have a major role in the prevention or delay of initial steps of colorectal cancer. The potentially important role of cocoa and their phenolic compounds for colon cancer prevention was first demonstrated by Weyant *et al.* [66]. In this study with a genetic model of multiple intestinal neoplasia that spontaneously develops numerous intestinal tumors, the authors demonstrated that the cocoa flavonoid catechin added to a diet in concentrations of 0.1% and 1%, was able to diminish the formation of intestinal tumors by 75% and 71%, respectively. Mechanistic studies linked this effect to (+)-catechin-induced changes in integrin-mediated intestinal cell-survival signaling, including structural alteration of the actin cytoskeleton and decreased focal adhesion kinase (FAK) tyrosine phosphorylation. As one of the earliest changes in adenoma development, FAK has been implicated in the regulation of cell migration [67], suggesting that (+)-catechin could prevent the progression of initiated enterocytes into the adenoma stage.

More recently, the chemo-preventive ability of a cocoa rich-diet on colon inflammation and pre-neoplastic lesions has been studied in male Wistar rats using the AOM-induced colon cancer model [44]. Administration of the colon-specific carcinogen AOM to rodents evokes the growth of aberrant crypt foci, which are pre-neoplastic lesions in the colon that may progress into cancer [68]. Feeding animals with a 12% cocoa-enriched diet for eight weeks suppressed intestinal inflammation induced by AOM through the inhibition of NF-κB signaling and the down-regulation of the expression of pro-inflammatory enzymes COX-2 and iNOS [44]. Moreover, cocoa flavanols indirectly reduced intestinal inflammation through their antioxidant and anti-proliferative effects. Thus, cocoa feeding was able to prevent oxidative stress by reverting the AOM-induced diminished levels of reduced glutathione and activities of GPx, GR and GST to basal values. Interestingly, in the same rat model of AOM-induced colon inflammation-cancer, a diet supplemented with dark chocolate has been reported to reduce cell proliferation and downregulate transcription levels of COX-2 and RelA resulting in a lower number of early pre-neoplastic lesions [69]. These results are summarized in Table 3.

## 4. Studies in Humans

Although substantial laboratory data from cell culture and animal studies support the anti-inflammatory effect of cocoa, extrapolation of the results to the human situation is difficult. It should be considered that animal experimental models are subjected to highly controlled conditions whereas exposure of humans to doses of pro-inflammatory compounds and other irritants may occur constantly and life-long. Additionally, the response of humans in an inflammatory situation to chemo-protective agents and diets may be strongly influenced by genetic polymorphisms, changes in DNA methylation and epigenomic events [70]. Thus, studies focusing on the effect of a regular consumption of cocoa or its derivatives on inflammatory markers, either related to cardiovascular disease or other pathologies such as cancer, are needed.

Many environmental causes and risk factors of cancer are associated with some form of chronic inflammation which acts as a driving force in the carcinogenic transformation of cells [7]. There are no proper human intervention studies attempting to show a correlation between cocoa intake and inflammatory markers for cancer prevention, but a few human intervention trials indicate that cocoa favorably affects intermediary factors of inflammation in cancer progression [71]. However, whether modification of any of these parameters will correlate with a lower incidence or development of specific cancers is highly debatable. A recent comprehensive review of the anti-inflammatory effect of cocoa flavanols associated with human cancer was written by Martín *et al.* [36].

### 4.1. Cocoa and Human Cardiovascular Disease

Inflammation is an important contributor to the development of endothelial dysfunction and atherosclerosis and inflammatory mediators appear to play a key role in each step of human atherogenesis, starting from the initial phases of leukocyte recruitment to the eventual rupture of the vulnerable atherosclerotic plaque [2]. Indeed, inflammation is considered a risk factor for human cardiovascular disease [2]. The accumulated evidence on the human health benefits of cocoa for disorders of a chronic inflammatory nature such as cardiovascular disease has been recently and thoroughly reviewed by Khan and colleagues [2]. Ever since some new studies have conferred a modest support to the anti-inflammatory effect of cocoa as well as confirmed the difficulty to achieve clear cut conclusions from human studies (Table 4).

#### 4.1.1. Cocoa and Inflammatory Interleukins and CRP

In support of the benefits of cocoa flavanols preventing endothelial inflammation and dysfunction, a recent report has shown that consumption of EC-containing apple puree increased NO metabolite status and attenuated platelet reactivity in healthy subjects (Table 4) [72]. Also recently, a randomised, cross-over, free-living study was carried out by Sarriá *et al.* [73] in 24 healthy and 20 moderately hypercholesterolaemic subjects to assess the influence of regularly consuming (four weeks) two servings (15 g each) of a cocoa product rich in fiber (containing 33.9% of total dietary fiber and 13.9 mg/g of soluble polyphenols) in milk *vs.* consuming only milk (Table 4). In those conditions, cocoa intake evoked an increase in serum HDL-cholesterol levels and a decrease in serum glucose, IL-1β, and IL-10 levels. In human subjects, the expression of IL-10 has been demonstrated in both coronary arteries and atherosclerotic plaques, and higher serum levels of IL-10 have been shown in atherosclerosis patients compared with controls, suggesting that the levels of IL-10, as an anti-inflammatory molecule, may be elevated in response to the pro-inflammatory environment of atherosclerosis [74]. In agreement with this, in the study by Sarriá and coworkers, IL-10 levels were higher in the hypercholesterolaemic subjects than in the normocholesterolaemic subjects (Table 4). IL-10 is involved in the inflammatory response by the downregulation of the synthesis of other cytokines, such as IL-1β [75], which is in accordance with the statistically significant reduction of IL-1β levels observed by Sarriá *et al.* [73]. Regarding serum lipid profile and cytokine levels, similar results have been reported after a representative intake of a flavanol-rich soluble cocoa product by moderately hypercholesterolemic subjects [76] and of a dietary-fiber-rich cocoa product by a similar human population (Table 4) [77]. However, the most recent data in support of an association between cocoa flavanols and decreased risk of various cardiovascular conditions is the epidemiology study by Kwok and coworkers [78]. This article also includes a meta-analysis of nine separate studies and 157,809 participants that found that higher chocolate consumption was significantly associated with lower risk of coronary artery disease, stroke, and cardiovascular mortality.

However, results from very recent studies have challenged the anti-inflammatory effects of cocoa flavanols (Table 4). Dower *et al.* [79] have reported that supplementation of pure EC reduced endothelial selectin, a marker of endothelial dysfunction, but did not affect biomarkers of inflammation in pre-hypertensive adults. This latter result is in line with those from Grassi *et al.* [80], Muniyappa *et al.* [81] and Monagas *et al.* [82] that found no significant effects on markers of inflammation such as IL-6, CRP or intercellular cell adhesion molecule (ICAM) after consumption of flavanol-rich cocoa or chocolate. Finally, a pro-inflammatory effect of cocoa flavanols has also been reported. In a study of healthy subjects, chocolate consumption increased release of TNF-α, IL-1β and IL-10 in microbial-stimulated mononuclear blood cells as an important mechanism through which chocolate consumption may influence acne [83]. These results may reflect differences in dose of cocoa or chocolate flavanols or else subjects’ health status. It is worth noting that flavanol concentration largely vary among cocoa and chocolate products; milk chocolate, dark chocolate, and natural cocoa powder contain 3 mg/g, 14 mg/g and 40 mg/g of flavanols, respectively [84].

#### 4.1.2. Cocoa and Soluble Adhesion Molecules

Soluble adhesion molecules are early biomarkers of alterations in vascular function that indirectly indicate vascular inflammation and endothelial cell activation [2,3,4,5,6]. Divergent effects of flavanol-rich foods on cell adhesion molecule level have been described. In support of a down-regulation of adhesion molecules by cocoa flavanols, in a widely referred study [85], hypercholesterolaemic postmenopausal women who consumed a high-flavanol cocoa beverage (446 mg of total flavanols) for six weeks had significantly lower levels of VCAM-1 compared with those consuming a low-flavanol cocoa beverage (43 mg of total flavanols). EC and certain B-type procyanidin dimers, as well as their related metabolites, are pointed as the candidates for the effects of high-flavanol cocoa beverage, as they inhibit the activation of the oxidative stress-sensitive NF-κB, a known promoter of VCAM-1 expression [85]. Likewise, in a study on high-risk cardiovascular subjects [82], ICAM-1 levels were significantly decreased after consuming 40 g of cocoa powder with milk (495 mg of total polyphenols). More recently, in a study in overweight men, dark chocolate consumption decreased plasma concentrations of ICAM-1 and ICAM-3 (Table 4) [86]. The combination of lower numbers of leukocytes, decreased leukocyte adhesion molecule expression, and decreased plasma soluble adhesion molecules after 4 weeks of dark chocolate consumption point toward reduced leukocyte adherence and subsequently reduced activation of the endothelium, the initial state of atherosclerosis [86]. In conflict with all of the above, ICAM-1 values did not change in obese adults at risk for insulin resistance that consumed cocoa beverages containing 30–900 mg flavanol per day within a controlled diet for five days [87], and VCAM-1 levels also remained unaltered after a fiber-rich [73] or flavanol-rich [76] cocoa intake both in normo- and hypercholesterolemic subjects. The use of cocoa products with different phenolic composition in distinct human sub-population groups (healthy, hypercholesterolemic, obese, dyslipidemic, diabetic, *etc.*) accounts for some apparently contradictory findings and challenges potential conclusions.

#### 4.1.3. Cocoa Fiber and Theobromine

Although there is increasing evidence supporting an anti-inflammatory effect of cocoa polyphenols by way of decreasing serum CRP levels as well as different cytokines (IL-2, IL-5, TNF-α, TGF-β) and adhesion molecules, the absence of any measurable impact has also been reported [88,89]. As above, the use of different cocoa products with dissimilar phenolic composition in distinct population groups might be the cause for the conflicting findings. Moreover, flavonoids are not the only cocoa components with potential bioactivity; dietary fiber and methylxanthines, particularly theobromine, are interesting cocoa constituents that are receiving more attention lately. In line with this, epidemiologic observations show that cereal fiber, and mainly insoluble dietary fiber, offers protection from cardiovascular disease through modulation of anti-inflammatory biomarkers [90]. In an interventional study, Qi and coworkers [91] observed that high whole grain intake was associated with lower CRP and TNF-α levels among women with type 2 diabetes. Other authors have also reported an inverse association between whole grain intake and CRP concentrations [92,93,94,95]. These effects of whole grains have been attributed to the synergistic anti-inflammatory effects of dietary fiber, minerals, antioxidants, polyphenols and other phytonutrients present in whole grains. 

The effect of dietary fiber on anti-inflammatory biomarkers others than CRP, has been scarcely studied. Intake of a high-fiber diet is associated with lower plasma levels of pro-inflammatory cytokines, IL-6 and TNF-R2 [96], and administration of a dietary fiber-rich cocoa product evoked a decrease of serum IL-1β, and IL-10 levels [73,77]. Actually, the studies of Sarriá and colleagues are the only ones that have investigated the effect of cocoa fiber in inflammatory biomarkers in humans. Two other major outcomes from these unique studies should be highlighted since they could also affect endothelial function preventing inflammation, atherogenesis and cardiovascular disease; the regular consumption of dietary fiber-rich cocoa products provoked an increase of serum HDL-cholesterol and a decrease of fasting serum glucose.

The increase found in HDL-cholesterol in the previously mentioned study is in line with other results in similar populations after an intake of flavanol-rich cocoa [76], of cocoa powder with milk [97] and also in agreement with the results described by Jenkins and colleagues [98] who reported a significant increase in HDL-cholesterol after consumption of a cocoa bran (25 g dietary fiber/day) for two weeks by healthy subjects. However, in a previous open intervention trial among free-living, moderately hypercholesterolemic volunteers who consumed two servings of a fibre-rich cocoa product daily, to provide a total dietary fiber of 12 g/day, HDL-cholesterol was not significantly increased after eight weeks as compared with baseline values [99]. Interestintly, Neufingerl and coworkers [100] have reported that theobromine independently increased HDL-cholesterol in a two-centre, double-blind, randomized, placebo-controlled, design in healthy subjects, suggesting the cocoa methylxanthine was responsible for increased HDL-cholesterol in cocoa interventions. However, in the study by Sarriá *et al.* [77], a higher intake of methylxanthines in one of the tested products did not result in a significant increase of HDL-cholesterol as compared to the other cocoa product, suggesting methylxanthines are not fully responsible for the HDL-cholesterol enhancing effect.

On the other hand, the decrease of fasting serum glucose after intake of a dietary fiber-rich cocoa product [77] is in agreement with the significant decrease in fasting plasma glucose observed in a previous study with a different dietary fiber-rich cocoa product [99]. Overall, polyphenol rich cocoa products have been shown to reduce fasting and postprandial blood glucose as well as insulin resistance [80,101,102], but contradictory results have also been published. This was evident in a study in obese adults at risk for insulin resistance where cocoa beverages containing 30–900 mg flavanol per day within a controlled diet for five days reduced inflammatory markers but did not affect glucose homeostasis parameters [87], and a minor but significant increase in fasting plasma glucose was observed in overweight men after four weeks of dark chocolate intervention [86]. Methylxanthines, particularly theobromine, may also have a role in lowering plasma glucose [103]. However, in the study by Sarriá *et al.* [77], out of two cocoa products containing a similar amount of flavanols and comparable levels of theobromine, only the product rich in dietary fiber evoked a significant decrease of fasting glucose, pointing to dietary fiber rather than theobromine as the cocoa constituent responsible for this hypoglycemic effect. 

#### 4.1.4. Cocoa in the Context of a Healthy Diet

A most interesting finding in recent human studies including cocoa interventions is that neither cocoa, nor chocolate induced any weight gain or other anthropometric changes [73,76,86]. Indeed, although cocoa products are usually high-energy foods, they have been shown to have anti-obesity effects in humans [104] and rats [32,56,57,105,106,107]. In addition, chocolate and other cocoa soluble product manufacturers are actively pursuing ways of producing novel low energy products by lowering sugar and fat levels without compromising the flavor and texture of traditional chocolate. In this line, it is worth mentioning that a recent study has shown that an increased flavanol content did not have an additional beneficial effect on markers of endothelial health, but did affect taste and had a negative effect on the motivation to eat chocolate [86].

Overall, despite some contradictory results, the fact is that cocoa, and especially its flavonoids, by way of their biological activities, including anti-inflammatory ones, show remarkable benefits in maintaining health and helping to prevent most significant diseases such as cardiovascular and cancer. Indeed, it is worth remembering that the European Food Safety Authority (EFSA) has aproved two claims in support of the bioactivity of cocoa flavanols: cocoa flavanols help maintain normal blood presure [108] and endothelium-dependent vasodilation [109], which contributes to normal blood flow. In order to obtain the claimed effect, 200 mg of cocoa flavanols should be consumed daily. This amount could be provided by 2.5 g of high-flavanol cocoa powder or 10 g of high-flavanol dark chocolate, both of which can be consumed in the context of a balanced diet. A recent review of current data has proved such a claim as the most optimal for prevention of cardiovascular disease [110].

## 5. Conclusions

A number of studies of cell cultures and animals have unequivocally demonstrated that cocoa flavanols reduce pro-inflammatory cytokines and inhibit inflammatory mediators NF-κB, COX-2 and iNOS. Several studies in animals and humans have additionally shown that cocoa intake or flavanol administration decrease critical biomarkers of endothelial inflammation such as VCAM and ICAM, further supporting the anti-inflammatory effect of cocoa. Disparity of some results may reflect differences in dose of cocoa or chocolate flavanols, cell culture origin and conditions, the animal model utilized or subjects’ health status.

Besides cocoa flavanols, in order to further deliniate the anti-inflammatory effect of a cocoa intake, the role of three compounds should be investigated: (i) host and microbiota-derived flavanol metabolites; (ii) cocoa fiber and (iii) theobromine. All three have shown promising beneficial effects on inflammation and cardiovascular health and they should be considered in future controlled clinical trials.

So far, the majority of the results from cell culture studies, animal experimentation and cocoa interventions in humans support the anti-inflammatory effect of cocoa compounds, especially flavanols, cocoa soluble products and cocoa-rich chocolates. Importantly, cocoa products are highly favored by the population and their recommended intake, especially for chocolate, can be consumed in the context of a balanced diet. 

## Figures and Tables

**Table 1:** Regulation by cocoa phenolic extracts and pure cocoa flavanols of proteins involved in the inflammatory process in cultured cells ^a^.

Polyphenol	Concentration	Effects	Reference
Cocoa polyphenol extract	50 µM	↓ PGE2	[43]
	5–80 µg/mL	↓ TNF-α, ↓ IL-1α, ↓ IL-6, ↓ LPS-induced TNF-α secretion, ↓ NO	[19]
	0.1–10 µg/mL	↓ PMA-induced superoxide production, ↓ IL-1α, ↓ IL-6	[36]
	10 µg/mL	↓ TNF-α-induced IL-8, COX-2, iNOS and NF-κB activation	[44]
	10–20 µg/mL	↓ TNF-α-induced NF-κB activation and PKB phosphorylation	[45]
	10 µg/mL	↓ LPS-induced NF-κB activation, IL-6, IL-1β	[48]
	50 µM	=LPS-induced IL-6, IL-8	[49]
Procyanidin polimers	2.5–60 µM	↓ TNF-α-induced NF-κB activation and iNOS	[41]
	10–25 µg/mL	↓ TNF-α-induced IL-8	[42]
Procyanidin B2	1.7–50 µM	↓ NF-κB binding, ↓ TNF-α and PMA-induced NF-κB activation (=PB1)	[36]
Epicatechin	200–400 µM	↓ MCP1, ↓ TNF-α, ↓ IL-1α, ↓ IL-6, ↓ NO	[19]
	1–10,000 µM	↓ LPS-induced nitrite and TNF-α production	[47]
	1.7–17.2 µM	↓ TNF-α-stimulated NF-κB	[36]
	10 µM	↑ NF-κB levels and nuclear translocation	[50]
Catechin	5–25 µg/mL	↓ LPS-induced nitrite and TNF-α production	[46]

^a^ The arrow indicates an increase (↑) or decrease (↓) in the levels or activity of the different analyzed proteins. PGE: prostaglandin E; PMA: phorbol 12-myristate 13-acetate; TNF: tumor necrosis factor; IL: interleukin; LPS: lipopolysaccharide; COX: cyclo-oxygenase; NOS: nitric oxide synthase; NF-κB: nuclear factor kappa B; PKB: protein kinase B; MCP: monocyte chemoattractant protein.

**Table 2:** Summary of recent data of the effects of cocoa and cocoa flavanols on inflammation markers in animal models of high-risk cardiovascular disease.

Induced Damage	Experimental Animal	Intervention	Duration	Main Outcomes ^a^	Reference
Obesity-related inflammation	C57BL/6 J mice	5% Cocoa-enriched diet (50 mg flavanols/Kg.b.w.)	18 weeks	↓ High fat diet-induced IL-6 and expression of pro-inflammatory genes	[56]
Adipose tissue inflammation	C57BL/6 J mice	5% Cocoa-enriched diet (50 mg flavanols/Kg.b.w.)	18 weeks	↓ High fat diet-induced TNF-α, IL-6, iNOS, NF-κB, COX-2	[57]
Adipose tissue inflammation	Swiss mice	10% Cocoa-enriched diet (50 mg flavanols/Kg.b.w.)	4 weeks	↑ High fat diet-down regulated PPARα expression and signaling	[58]
Experimental myocarditis	Balb/c mice	Cocoa polyphenol extract (oral)	3 weeks	↓ Myocarditis-induced IL-1β, IL-6, E-selectin, VCAM-1, NF-κB	[60]
Atherogenic diet	ApoE3Leiden mice	High flavanol chocolate-enriched diet (8.75%)	20 weeks	↑ High cholesterol-induced VCAM-1 and E-selectin	[62]

**^a^** The arrow indicates an increase (↑) or decrease (↓). PPAR: Peroxisome Proliferator-Activated Receptor; TNF: tumor necrosis factor; IL: interleukin; COX: cyclo-oxygenase; NOS: nitric oxide synthase; NF-κB: nuclear factor kappa B; VCAM: vascular cell adhesion molecule.

**Table 3:** Summary of effects of cocoa, cocoa flavanols and cocoa derivatives on inflammation markers in animal models of colon inflammation and cancer.

Induced Damage	Experimental Animal	Intervention	Duration	Main Outcomes ^a^	Reference
Ulcerative colitis	Mice	Cocoa polyphenol extract oral (30 mg flavanols/Kg.b.w.)	7 days	↓ DSS-induced NO, COX-2, pSTAT-3, pSTAT1R, NF-κB	[48]
Ulcerative colitis	Wistar rats	5% Cocoa-enriched diet (30 mg flavanols/Kg.b.w.)	2 weeks	↓ DSS-induced TNF-α and iNOS	[54]
Chronic inflammation	BALB/c mice	5%–10% Cocoa-enriched diet (9–18 mg flavanols/Kg.b.w.)	8 weeks	↓ DSS/AOM-induced iNOS and COX-2	[64]
Chronic inflammation	BALB/c mice	5%–10% Cocoa-enriched diet (9–18 mg flavanols/Kg.b.w.)	8 weeks	↓ DSS/AOM-induced IL-6	[65]
Colon cancer	Female C57BL/6J-Min/1 mouse	(+)-Catechin (0.1% or 1%)	10 weeks	↓ FAK tyrosine phosphorylation and tumour number	[66]
Preneoplastic lesions	Wistar rats	Cocoa-rich diet (12%)	8 weeks	↓ AOM-induced NF-κB and COX-2 and iNOS expression	[44]
Preneoplastic lesions	Rats	Dark chocolate (72 mg flavanols/Kg.b.w.)	_	↓ AOM-induced COX-2 and RelA	[69]

^a^ The arrow indicates an increase (↑) or decrease (↓). AOM: azoxymethane.; DSS: dextran sulfate sodium; FAK: focal adhesion kinase; TNF: tumor necrosis factor; IL: interleukin; COX: cyclo-oxygenase; iNOS: inducible nitric oxide synthase; NF-κB: nuclear factor kappa B; pSTAT: phosphorilated signal transducer and activator of transcription protein.

**Table 4:** Summary of most recent effects of cocoa and cocoa flavanols on anti-inflammatory markers in human cohorts.

Subjects	Experimental Design	Intervention	Duration	Main Outcomes ^a^	Reference
Healthy adults	Randomized, three-phase crossover	High-EC apple pure (25–100 mg)	4 weeks	↑ NO metabolite concentration ↓ P-selectin expression	[72]
Moderately hypercholesterolaemic	Randomised, controlled, cross-over, free-living	Fiber-rich cocoa soluble (416 mg flavanols/day)	4 weeks	↓ IL-1β, IL-10, = VCAM1	[73]
Moderately hypercholesterolaemic	Randomised, controlled, cross-over, free-living	Flavanol-rich cocoa powder (45 mg flavanols/Kg.b.w.)	4 weeks	↓ IL-10, = VCAM1	[76]
Moderately hypercholesterolaemic	Randomised, controlled, cross-over, free-living	Fiber-rich cocoa soluble (44 mg flavanols and 10 g dietary fiber/day)	4 weeks	↓ IL-1β	[77]
Healthy (pre)hypertensive	Randomized, doubleblind, placebo-controlled crossover trial	EC (100 mg/day)	4 weeks	↓ sE-selectin, = IL-1β	[79]
Overweight men	Randomized doubleblind Crossover	High-flavanol chocolate (70 g/day)	4 weeks	↓ sICAM1 and sICAM3	[86]

^a^ The arrow indicates an increase (↑) or decrease (↓). IL: interleukin; VCAM: vascular cell adhesion molecule; ICAM: intercellular cell adhesion molecule.
